# Automated proximity sensing in small vertebrates: design of miniaturized sensor nodes and first field tests in bats

**DOI:** 10.1002/ece3.2040

**Published:** 2016-03-02

**Authors:** Simon Ripperger, Darija Josic, Martin Hierold, Alexander Koelpin, Robert Weigel, Markus Hartmann, Rachel Page, Frieder Mayer

**Affiliations:** ^1^ Museum für NaturkundeLeibniz Institute for Evolution and Biodiversity ScienceBerlinGermany; ^2^ Institute for Electronics EngineeringFriedrich‐Alexander‐Universität Erlangen‐Nürnberg (FAU)ErlangenGermany; ^3^ Information Technology (Communication Electronics)Friedrich‐Alexander‐Universität Erlangen‐Nürnberg (FAU)ErlangenGermany; ^4^ Smithsonian Tropical Research InstituteApartado0843‐03092Balboa, AncónPanama; ^5^ Berlin‐Brandenburg Institute of Advanced Biodiversity ResearchBerlinGermany

**Keywords:** Automated data collection, digital telemetry, encounter logging, sensor network, social interactions

## Abstract

Social evolution has led to a stunning diversity of complex social behavior, in particular in vertebrate taxa. Thorough documentation of social interactions is crucial to study the causes and consequences of sociality in gregarious animals. Wireless digital transceivers represent a promising tool to revolutionize data collection for the study of social interactions in terms of the degree of automation, data quantity, and quality. Unfortunately, devices for automated proximity sensing via direct communication among animal‐borne sensors are usually heavy and do not allow for the investigation of small animal species, which represent the majority of avian and mammalian taxa. We present a lightweight animal‐borne sensor node that is built from commercially available components and uses a sophisticated scheme for energy‐efficient communication, with high sampling rates at relatively low power consumption. We demonstrate the basic functionality of the sensor node under laboratory conditions and its applicability for the study of social interactions among free‐ranging animals. The first field tests were performed on two species of bats in temperate and tropical ecosystems. At <2 g, this sensor node is light enough to observe a broad spectrum of taxa including small vertebrates. Given our specifications, the system was especially sensitive to changes in distance within the short range (up to a distance of 4 m between tags). High spatial resolution at short distances enables the evaluation of interactions among individuals at a fine scale and the investigation of close contacts. This technology opens new avenues of research, allowing detailed investigation of events associated with social contact, such as mating behavior, pathogen transmission, social learning, and resource sharing. Social behavior that is not easily observed becomes observable, for example, in animals living in burrows or in nocturnal animals. A switch from traditional methods to the application of digital transceiver chips in proximity sensing offers numerous advantages in addition to an enormous increase in data quality and quantity. For future applications, the platform allows for the integration of additional sensors that may collect physiological or environmental data. Such information complements social network studies and may allow for a deeper understanding of animal ecology and social behavior.

## Introduction

Animal social systems show a stunning diversity in terms of organization, mating systems, and social structure. Here, social structure is defined as the sum of all dyadic relationships among the members of a social group which are in turn defined by the quality of interactions (Kappeler et al. 2013). Social interactions among conspecifics may show considerable plasticity. Their frequency and intensity may vary seasonally as a consequence of environmental conditions (Robert et al. 2013; Williams et al. 2013) or may be restricted to certain geographic locations by the joint use of a site, for example, roosts or latrines, while foraging is performed solitarily (Kerth et al. 2001; Dröscher and Kappeler 2014). Social interactions are the basis for complex behaviors such as decision making (Conradt and Roper 2005; Couzin et al. 2005) or transmission of knowledge in groups (van Schaik and Burkart 2011). In consequence, thorough documentation of social contacts among individuals in gregarious animal species can provide another piece in the puzzle of understanding the evolution of sociality.

Direct observation is a common method used to keep track of individual interactions among terrestrial group‐living animals. However, this method is labor‐intensive and not feasible in practice for species with cryptic lifestyles. Technical aids allow for remote data collection at low levels of disturbance. Classic wildlife tracking technology (for instance, VHF‐transmitters or GPS‐collars) enables researchers to colocalize individuals and to study, for example, group hunting, social bonds, or decision making in group‐living animals (de Melo et al. 2007; Dechmann et al. 2010; Strandburg‐Peshkin et al. 2015). However, indirect documentation of interactions via colocalization generates considerably less accurate data sets than direct encounter mapping via proximity loggers (Krause et al. 2013). Proximity loggers (e.g., in the form of collars by “Sirtrack: Wildlife Tracking Solutions”) quantify the time collars are less than a user‐defined distance from one another (Boyland et al. 2013). Interactions between individuals can thus be documented for long periods of time (Marsh et al. 2011). The resulting contact networks allow conclusions to be drawn on a wide variety of social interactions in nature, for example, on the potential transmission of infectious diseases among individuals of wild‐living species (Böhm et al. 2009) and between domesticated animals and wildlife (Noer et al. 2012). The most sophisticated proximity loggers available are digital transceivers developed during the “Encounternet project” (in the following referred to as “Encounternet”) (Mennill et al. 2012). Modified versions of Encounternet allow for mapping of social systems and documentation of mating behavior in birds at unprecedented spatiotemporal resolution by the communication of animal‐borne transceivers to either stationary receivers (Snijders et al. 2014; Maynard et al. 2015) or in addition among the transceivers themselves (Rutz et al. 2012; St Clair et al. 2015).

The described devices for direct encounter mapping still impose restrictions to research. Sirtrack proximity loggers are able to communicate directly to several other loggers at a time. However, the gathered information is of binary nature because the encounter is considered ongoing as long as the loggers do not exceed the user‐defined distance (Boyland et al. 2013). Fine‐grained changes as a result of variable distances among individuals, which may help to interpret the quality of an interaction (ranging from, e.g., being in direct body contact to being several meters apart), cannot be monitored. Furthermore, the loggers are quite heavy (20–150 g) and need to be retrieved for data recovery via an emitted VHF signal (Böhm et al. 2009; Hamede et al. 2009; Marsh et al. 2011). Encounternet overcame some of these limitations by full automation for data collection and acquisition at a transceiver weight of <10 g (Rutz et al. 2012). Even more remarkable, it documents a “received signal strength indicator” (RSSI) value of each encounter. These data can be used to interpret the animal‐to‐animal distance after thorough system calibration and have been applied to study social networks in New Caledonian crows at unprecedented resolution (Rutz et al. 2012, 2015; St Clair et al. 2015). The most recent version of Encounternet tags weighs as few as 1.3 g and was used to study social networks in barn swallows (Levin et al. 2015). This weight reduction comes at the expense of battery life which limits data collection to approximately 21 h. However, scheduling capabilities that restrict data collection to the activity periods of the focus animals enable the extension of the operation time to several days.

Direct encounter mapping creates richer and more accurate data sets on interactions among animals, but the considerable size of most available loggers has restricted the application to medium‐ to large‐bodied vertebrates in the past (Krause et al. 2013). Sophisticated proximity loggers are crucial for the construction of weighted social networks. Therefore, research in the establishment of proximity loggers for small vertebrates is necessary in order to advance our understanding of sociality in animals (Ryder et al. 2012). Here, we present a low‐weight, energy‐efficient sensor network that consists of animal‐borne (mobile) nodes and stationary ground nodes. Mobile nodes are able to document signal strength values which were received from other mobile nodes and are forwarded to ground nodes. Ground nodes automatically receive data packages from mobile nodes and document signal strength values received from mobile nodes. A sophisticated scheme for energy‐efficient communication allows for high sampling rates at relatively low power consumption. At <2 g, the mobile nodes can be carried by a broad spectrum of taxa including small vertebrates. We present the system architecture, an evaluation of sensor performance under standardized conditions, and verification of the system's functionality to monitor wildlife exemplarily on a highly cryptic animal taxon: bats.

## Material and Methods

### General functionality of the system

The two main components of the system were animal‐borne mobile nodes and stationary ground nodes (see Box 1). The most critical issue in designing a lightweight mobile node was to insure an efficient power management because the battery contributed the largest portion to overall weight. We reduced power consumption on the mobile nodes by realizing a time‐slotted communication scheme, which is much less energy intense than permanent transmission and reception. Each mobile node sent out a unique ID within a dedicated time slot while the remaining mobile nodes (and the ground node) listened to receive a transmitted signal. The tags' communication was scheduled via internal clocks that needed to be synchronized to a global time reference (Hierold et al. 2015a) which was broadcasted by a transmitter on the ground node. If a mobile node received the ID from another mobile node, the RSSI and an absolute timestamp of the encounter were stored in a ring buffer and transmitted to a receiver on the base station along with the IDs of the mobile nodes in contact. Consequently, proper operation could only be achieved when the tags were in communication range of a ground node. The ground node communication range was approximately 30 m in vegetated environments. The use of multiple base stations allowed for covering larger areas (for details see Hierold et al. 2015b).

Box 1 1 *Ground node*: a stationary transceiver that writes received data gathered from mobile nodes to a flash memory and assigns time slots to mobile nodes for communication. Time reference on ground nodes is obtained by an integrated GPS receiver.

*Mobile nodes*: animal‐borne transceivers that create encounter log when they are in communication range with other mobile nodes and forward stored logs when in communication range with ground nodes. The terms “mobile node” and “tag” are used synonymously.

*Log*: protocol listing of an encounter event among two mobile nodes which is created on either mobile node. A log includes the IDs of the involved mobile nodes, ID of the ground node, timestamps on the mobile and the ground node, the RSSI value from the encountered mobile node and the RSSI values from the ground node.

*Meeting*: consecutive logs add to meetings. We considered meetings ongoing when consecutive logs were not separated in time for longer than 5 sec.

*RSSI*: Received Signal Strength Indicator. RSSI values serve as proxies in order to estimate distances among communicating nodes.

*dBm*: decibels relative to one milliwatt, an electrical power unit in decibels (dB).

### Design for tests of proximity sensor prototypes

We chose a three step design with increasing complexity for testing the functionality of prototypes starting (i) under predefined laboratory conditions, followed by tests on live bats, (ii) inside their roost, and (iii) outside the roost.

- For performance tests and calibration, we documented RSSI values for several combinations of prototypes. We attached the tags to balloons that were filled with 20 mL of water in order to mimic the attachment to a small animal. We tested the variation of received signal strength depending on the distance between two mobile nodes at fixed distance intervals ranging from 0.5 to 4 m. Furthermore, we documented the influence of the antenna alignment (parallel, orthogonal, in line) among mobile nodes on RSSI values.
- The functionality of prototypes on live animals was verified in a maternity colony of free ranging greater mouse‐eared bats *Myotis myotis* (Borkhausen) that was located inside an attic. The aim of this test was to validate the proper communication among mobile and ground nodes which indicates the presence of tagged individuals inside the roost. Second, we related changes in the distance among tagged individuals from video footage within the colony to variation in received signal strength among mobile nodes.
- The goal of the final field test was to document meetings of individual bats after they have left their roost for foraging. For this purpose, we worked on social groups of the fringe‐lipped bat *Trachops cirrhosus* (Spix).

In the following, we use (i), (ii), and (iii) in order to refer to the respective test designs.

### Mobile node designs

**Figure 1:**
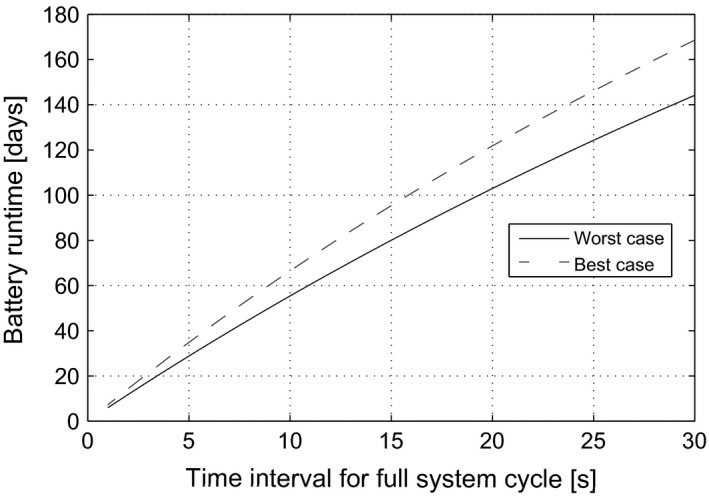
Relationship between the time needed for a full system cycle of six mobile nodes and operation time. Worst case indicates maximum data traffic in case of permanent contact to a ground node. Best case indicates that no data are sent to the ground node, which increases operation time because of lower data traffic.

The mobile nodes based on the System on Chip solution CC430 from Texas Instruments comprising a microcontroller and a subgigahertz frontend (868 MHz (ii), 902.2 MHz (iii)). The tags were powered by a lithium coin battery. A 330 *μ*F buffer capacitance was applied in parallel to the battery in order to facilitate the drawing of the high current peaks necessary during the active phases of the tag (Fig. S1 shows the hardware architecture of the mobile node). A full system cycle which passed through the time slots for ID transmission of five tags lasted 2.1 sec (ii) or 1 sec for six tags (iii), respectively. Theoretically, any number of mobile nodes may be used in an experimental setup. However, the time needed for a full system cycle will increase and the temporal resolution of the obtained data will decrease accordingly. A matching network connected the chip's output pins for signal emission to a whip antenna. Finally, tags were sealed with epoxy resin in order to protect tags from damage by, for example, exposure to humidity or manipulation by the bats. The mobile nodes were equipped with coin batteries of different sizes (either 0.6 g CR1025 battery, 30 mAh or 0.8 g BR1225 battery, 48 mAh), leading to a final weight between 1.4 and 1.9 g. Depending on the battery type and the communication scheme, the mobile nodes allowed for total operation times between 6 and 9 days (average current consumption 166 *μ*A [ii] and 270 *μ*A [iii]). The operation time of mobile nodes can be increased considerably by lowering the sampling rate, that is, by extending the time needed for a full system cycle (see Fig. 1 for a calculation based on the design described in [iii]). For example, an increase in the sampling interval of a full system cycle to 30 sec will extend the operation time to more than 140 days.

### Ground node designs

The ground nodes were composed of a dedicated transmitter and receiver for simultaneous signal transmission and reception. A low‐cost Olimex MSP430 CCRF development board was used to transmit and receive signals, respectively. The ground nodes were equipped with a Trimble GPS timing module (Resolution SMTx, Trimble Navigation, Ltd., Sunnyvale, California, USA) on a Carrier Card with a suitable GPS active antenna in order to guarantee the distribution of a precise global time reference. The received data were stored on a SD card where it was easily accessible at any point of the field study. Each ground node was powered by a lead‐gel battery that offered an operation time of 9.5 days (Fig. S2 shows the hardware architecture of the ground node). The ground nodes were protected by plastic housings from environmental influences.

### Focus species and study sites of field tests on live bats

The first field test of the system to monitor live animals (ii) was conducted in a maternity roost of the greater mouse‐eared bat (*M. myotis*) in Leutenbach, Bavaria (49°42′30″N, 11°10′27″E). Females of this temperate bat species form aggregations in large buildings where they give birth and rear their young, while males usually roost solitarily (Petri et al. 1997). The colony we studied inhabited the attic of a rectory and a census in July 2014 indicated the presence of 154 individuals including offspring and adult females (M. Hammer, Coordination Unit for Bat Conservation in Northern Bavaria, pers. comm.). The colony offspring were already flying independently when our observations started.

The second field test (iii) was conducted at the Smithsonian Tropical Research Institute in Gamboa, Panama (09°07′N, 79°42′W) and focused on social groups of the fringe‐lipped bat (*T. cirrhosus*). *Trachops cirrhosus* roosts in mixed sex groups of variable size (Cramer et al. 2001). In central Panama, fringe‐lipped bats can be seen foraging at ponds where they hunt for calling frogs (Tuttle and Ryan 1981). Foraging areas are usually rather small and repeatedly visited by individual bats over a series of nights (Kalko et al. 1999).

### Animal capture and deployment of mobile and ground nodes

We caught bats with monofilament nets (Ecotone, M‐series) while they were leaving their roosts. All captured individuals were sexed, and weight, size of forearm, age, and reproductive state were documented. Mobile nodes were glued to the skin between the scapulae with Permatype Surgical cement after partially trimming the fur. For field test (ii), we equipped four adult females of *M. myotis* with mobile nodes on 21 July 2014 (Table 1) and installed one mobile node inside the attic as a known, permanently present reference. The communication among the mobile nodes was synchronized by one ground node that was also deployed inside the attic. Data were collected until 25 July. For field test (iii), we tagged four individuals of *T. cirrhosus* from a social group (IDs 5–8, Table 1) on 4 February 2015 and five individuals from a second group on 7 February 2015 (IDs 9–13). The observation lasted six days, respectively. A total of six ground nodes were deployed in proximity to the day roosts and to standing water bodies in order to observe potential meetings among bat individuals when the bats leave or return to the roost or during foraging.

**Table 1:** Summary of physiological and tag parameters of 13 tagged bats from two species (Mmyo: *Myotis myotis*; Tcir: *Trachops cirrhosus*). Reproductive state: NR = nonreproductive, LAC = lactating, R = reproductive male

ID	Species	Sex	Age	Reproductive state	Forearm (mm)	Body weight (g)	Transmitter weight (g)	Body/transmitter weight ratio (%)
1	Mmyo	F	AD	NR	62.2	27	1.4	5.2
2	Mmyo	F	AD	NR	62.7	25	1.8	7.2
3	Mmyo	F	AD	NR	60.8	25	1.8	7.2
4	Mmyo	F	AD	LAC	64.3	27	1.8	6.7
5	Tcir	F	AD	NR	61.7	40	1.8	4.5
6	Tcir	M	AD	NR	55.9	31	1.7	5.5
7	Tcir	M	AD	R	57.2	33	1.8	5.4
8	Tcir	F	AD	NR	60.0	34	1.9	5.6
9	Tcir	M	AD	NR	59	34.5	1.8	5.2
10	Tcir	F	AD	LAC	60.4	36	1.8	5.0
11	Tcir	F	J	NR	60.2	28	1.8	6.4
12	Tcir	F	J	NR	59.4	29	1.8	6.2
13	Tcir	F	AD	LAC	56.9	45	1.8	4.0

### Video recordings of the nursing colony of *M. myotis*

We videorecorded the nursery colony of *M. myotis* in order to verify the presence of tagged bats and to relate changes in signal strength among bat‐borne mobile nodes to displacements of the tagged bat individuals. We used a Sony night shot camcorder (DCR‐TRV8E) which was supported by a 48 LED infrared spotlight. The camcorder was connected to an Apple MacBook Pro via firewire, which digitally captured the material in iMovie 8.0.6 (Apple Inc., Cupertino, California, USA). The video footage was then surveyed for the presence of bats that carried the sensor nodes. Individual identification of the tagged bats was possible by differently shaped pieces of reflector tape, which were glued to the mobile nodes.

### Data analysis

The data that were generated by mobile and ground nodes were forwarded to a PC and written to text files by the MATLAB R2013a software (MathWorks, Natick, Massachusetts, USA; [ii]). Distances between individually tagged *M. myotis* were measured from screenshots from video footage in ImageJ 1.48v (Schneider et al. 2012). In Panama (iii), the data were written to text files and stored on SD cards directly on the ground nodes.

We fitted linear mixed‐effects models in R 2.15.1 (R Core Team 2015) using the library “nlme” (Pinheiro et al. 2012) in order to test for a relationship between RSSI values obtained in laboratory experiments (i) and distances between two mobile nodes. We modeled signal intensity (RSSI) as the response variable and we added distance between communicating nodes as a fixed effect. We included the ID combination of communicating tags as a random effect in order to control for repeated sampling of several tag combinations. Marginal and conditional *R* ^2^‐values were calculated in the library “MuMIn” (Barton 2013). Finally, we applied Tukey's honest significance post hoc test using the R library “multicomp” (Hothorn et al. 2008). We used the Wilcoxon test for paired samples in order to test for differences in RSSI values between two bat‐borne mobile nodes before and after a displacement inside the *M. myotis* maternity roost (during field test [ii]).

## Results

### 1 Functionality of nodes under predefined conditions

**Figure 2:**
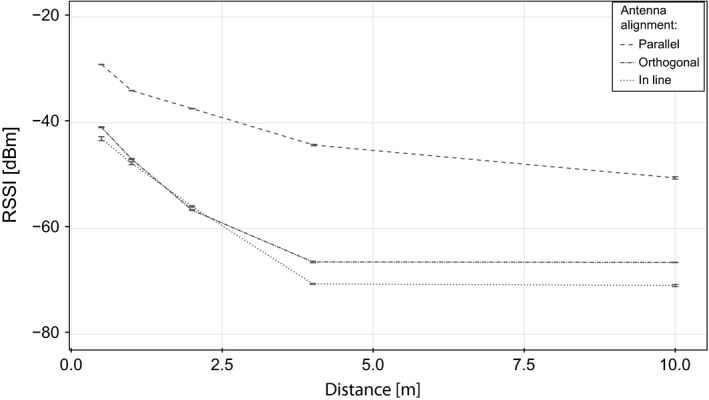
Received signal strength (RSSI) dependent on distance and antenna alignment between mobile nodes. RSSI values were documented for parallel, linear, and orthogonal alignment of mobile nodes' antennas at 0.5, 1, 2, 4, and 10 m distance.

We observed considerable variation in received signal strength (RSSI) depending on both the alignment of the antennas of two communicating nodes at a particular distance and the distance between two mobile nodes. The signal propagation was similar to the free space loss when the antennas were aligned perfectly in parallel (Fig. 2). Orthogonal or serial antenna alignments between the transmitting and the receiving node caused signal attenuation but did not cause signal loss. We received reliable signals up to a distance of at least 10 m. An orthogonal or serial alignment caused an intensity drop of more than 10 dB compared to parallel antenna alignment in close distance of 0.5 m. With increasing distance of up to 10 m, we observed an intensity drop of 15 dB and more.

**Figure 3:**
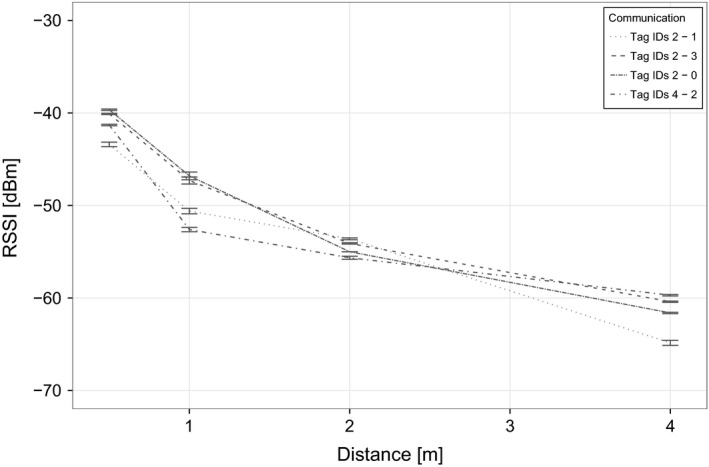
Distance dependence of the received signal strength (RSSI) for four different tag combinations. Tag IDs 1–4 refer to IDs in Table 1.

Received signal strength indicator values of parallel aligned antennas decreased significantly with increasing distance (Fig. 3; mixed linear model, effect of distance: *F*_3,151_ = 1225, *P* < 0.0001). Pairwise comparisons revealed a significant decrease of received signal intensity with every stepwise increase in distance (Tukey test: 0.5–1 m, 1–2 m, 2–4 m, *P* < 0.0001 for all comparisons). Still, interpair variation could reach 5–7 dB depending on the distance among tags.

### 2 Functionality of nodes on live bats inside the roost

The system reliably documented the presence of the tagged bats. On 22 July, the day after the bats were tagged, only bat 4 was present in the colony. Bats 1, 2, and 3 were inside the attic during daytime on 23 and 25 July. On 24 July, only bat 3 was present. Observations during the daily inspection of the colony and a survey of video footage confirmed the data collected by the sensor system.

**Figure 4:**
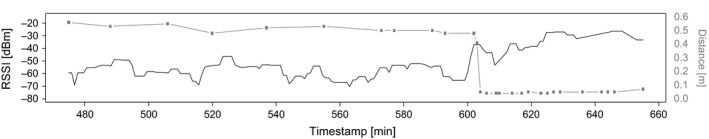
Variation in received signal strength (RSSI) between the bats ID 1 and ID 2 over time (black solid line). RSSI values per timestamp are displayed as a maximum value per 3 min interval. The distance between the bats ID 1 and ID 2 over time is shown by the gray broken line.

We documented received signal strength between the tagged individuals whenever more than one bat individual was present in the colony. This was possible over the course of the days of 23 July and 25 July when individuals 1, 2, and 3 were present simultaneously. Survey of video footage allowed for linking sustained changes in signal strength to bat movement. We observed individual 2 after being at a rather fixed distance of ca. 50 cm from individual 1 moving very close toward the latter. This displacement was accompanied by a significant increase in RSSI intensity (Fig. 4; Wilcoxon test, *W* = 62890.5, *P* < 0.001).

A comparison of Figs. 3, 4 shows that RSSI values at a distance between bats 1 and 2 of ca. 50 cm may come close to the values obtained for tag combination 1–2 under laboratory conditions in parallel antenna alignment, but usually RSSI values among animal‐borne nodes stay below those collected in the laboratory.

### 3 Functionality of nodes on live bats outside the roost

**Figure 5:**
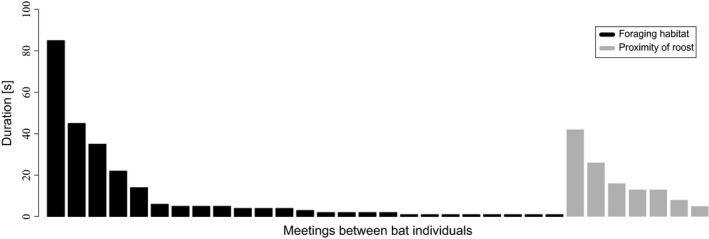
Durations of meetings for four dyads of bats from two social groups of *Trachops cirrhosus*. Meetings were documented in close proximity to the day roosts or standing water bodies.

We documented meetings among four bat dyads. In total, 467 encounter logs were created and successfully forwarded to the ground nodes. These logs contributed to 32 meetings and a meeting was considered ongoing when no gaps longer than 5‐sec were present. For one social group of *T. cirrhosus* (IDs 5–8), only one meeting was recorded. Individuals 7 and 8 were in communication range for a time span of 25 sec in proximity to the roost. Three dyads from the second social group (IDs 9–13) contributed to 31 meetings that originated from 437 logs. Most logs originated from 22 meetings between an adult, lactating female (ID 10), and a juvenile female (ID 11). Six meetings were documented between individual 11 and another juvenile female (ID 13). Bat 13 met bat 10 three times. The durations of the meetings were highly variable. The shortest meetings that were documented lasted 1 sec, the lower bound in time resolution of the system. The longest meeting lasted 85 sec and consisted of 74 logs (Fig. 5). The meetings occurred either in close proximity to the roost or to standing water bodies.

### Observations on welfare

In *M. myotis,* we observed the positions of the tagged animals within the group throughout the experiment in order to assess whether the sensor nodes or the antennas hindered the bats in joining the tight clusters which are typical for this species (Zahn 1999). Whenever tagged individuals were observed on video or during daily visits of the colony, they were roosting within large clusters and never isolated. During a census and mist‐netting in July and August 2015, all four tagged bats were rediscovered.

For *T. cirrhosus*, we also observed no ill effects in response to the tags. Of the nine individuals tracked for this study, five were recaptured in the same roosts 1–5 months later. All recaptures were in excellent health, with no drop in weight, and no noticeable effects in response to the tags.

## Discussion

Commercially available proximity loggers, capable of tag‐to‐tag communication enabling the study of group dynamics at high resolution, weighed roughly 10 g or even tens of grams up until very recently. Therefore, limited data are available on weighted social networks of small animals (Ryder et al. 2012; Levin et al. 2015) because tag weight determines the observable animal spectrum and limits application in small animals (Cooke et al. 2004). Future advances in battery technology would promote the miniaturization of tags (Rutz and Hays 2009; Levin et al. 2015). However, this breakthrough is not yet in sight. A possible workaround is to reduce the tags' energy demand by scheduling the data collection to the main activity periods of the focus animals. Levin et al. (2015) present such an approach that allows for data collection over at least 3 days at a tag weight of only 1.3 g. Our system circumvents the issue of high energy demand (and the associated battery load) by a sophisticated communication pattern that ensures high sampling rates at relatively low power consumption. This strategy safeguards low weight of tags of <2 g and operation times of at least 6 days even at very high sampling rates. Tags within this particular size class will finally allow us to study the most species‐rich body weight classes in mammals and birds (Kays et al. 2015).

Our miniaturized transceiver chips performed robust communication both between mobile nodes and among mobile and stationary nodes under laboratory conditions as well as when attached to free living bats. During the observations on the maternity colony, the signal strength of the tagged individuals received at the base station was subject to rather strong fluctuations. In the course of the day, variations may be the results of displacements of the group in consequence of microclimatic changes (Zahn 1999). Strong fluctuations in the short term most likely originated from permanent turning and jiggling that could be observed on video for most individuals of the colony. This restless behavior causes continuous changes in antenna alignment among mobile and ground nodes or even temporary buckling of antennas by close body contact among colony members. The received signal strength responded rather strongly to nonideal antenna alignment in test trials. Post hoc data filtering offers opportunities to mitigate the problems that arise from active, moving animals. The extraction of a maximum RSSI value per fixed time interval increases the probability to encounter a quasi‐ideal alignment of antennas and will most likely reflect reality. RSSI values from two tagged bats that were spaced ca. 50 cm apart did occasionally come close to laboratory values. Such time intervals need to be chosen depending on the observed species, the research question, and the situation. For example, in our observation of bats, buckling of antennas or strong deviation from parallel alignment would rather be expected with large bat clusters within the roost than when observing, for example, tandem flights.

Proximity sensing is a promising tool for increasing both data quality and quantity, especially in the analysis of social networks (Ryder et al. 2012). The mobile nodes responded under both laboratory conditions and when carried by bats with significant changes in signal intensity to minor changes in distance among nodes. Given our specifications, the system was especially sensitive to changes in distance within a short range (up to 4 m). This high resolution at short distances allows for evaluating interactions among individuals at a finer scale than most proximity sensors which can only detect whether the distance between two tagged animals fell below a certain threshold or only communication between a tagged animal and a receiver station (Mennill et al. 2012; Boyland et al. 2013). In this way, our system is a valuable tool for tracking close contacts among individuals. This provides new opportunities to study events in detail that may be associated with social contacts such as transmission of pathogens, mating behavior, social learning, or resource sharing (Wey et al. 2008).

The most challenging part in analyzing proximity data is the conversion of RSSI values that are logged by animal‐borne tags into interanimal distances. It is practically impossible to achieve precise distance measures as a result of variability in signal attenuation over distance caused by, for example, variation in habitat types, antenna alignment, or multipath scattering. However, Rutz et al. (2015) present a comprehensive guide for the calibration of animal‐borne proximity loggers. Here, proximity loggers are calibrated following a protocol that is tailored to the specific research project. The distance–signal strength relationship needs to be documented thoroughly across habitats or vertical strata that are visited by the focus species and needs to be supplemented with statistical models and computer simulations. Admittedly, the presented approach is work‐intensive, but it allows for a robust post hoc estimation of interindividual distance categories based on variation in RSSI values. These categories are created for the particular research question and respond to biologically meaningful situations (St Clair et al. 2015).

Wireless digital transceiver technology has already proven to revolutionize data collection on animal interactions in terms of the degree of automation, data quantity, and quality (Rutz et al. 2012). Further tag miniaturization is crucial to gain a deeper understanding of the causes and consequences of the tremendous variety of social organizations, mating systems, and social structures in gregarious mammalian and avian taxa (Wrangham and Rubenstein 1986; Kerth 2008; Kappeler et al. 2013). Automated approaches using lightweight tags will permit access to a wider spectrum of animal species because the majority of mammal and bird species features small body weight or cryptic lifestyle (Kays et al. 2015). Until now, sociality has rarely been studied in small‐bodied taxa, such as bats, in detail, but it appears to be far more complex than previously thought (Kerth 2008). Our data show that encounters among group members are not restricted to roosts, but also occur during foraging – a behavior that is almost impossible to observe with conventional technology. Sensor technology can increase data quality in studies on group dynamics in fission–fusion societies or the process of information transfer among members of social groups. Until recently PIT tags with very short detection ranges (Patriquin et al. 2010; Burns and Broders 2015; Farine et al. 2015) or experimental setups in captivity (Page and Ryan 2006; Clarin et al. 2014; O'Mara et al. 2014) have been applied to study such behavior. Digital transceiver chips offer besides increased data quality the possibility to include additional sensors for documenting supplementary data on environmental factors. Both, high data quality and the integration of additional data types may help to understand the evolution of social behavior (Silk et al. 2014) or to test hypotheses on the evolution of social learning (van Schaik and Burkart 2011).

High‐resolution encounter data are not only the key to the understanding of group dynamics per se; it also gives insight into associated events, for example, the transmission of pathogens. A range of taxa of small vertebrates have been identified to carry pathogens which may partly infect humans (e.g., bats: Drexler et al. (2012), rodents: Vaheri et al. (2013), birds: Olsen et al. (2006)). Spread within animal populations does not only depend on population densities, which is often used as a proxy for encounter rates, it is also influenced by individual behavior (Keesing et al. 2010). The quality of interactions, for example, whether it comes to direct contact or not, may play a role in pathogen transmission (Schauber et al. 2015). A monitoring system that documents intensity and duration of encounters in small vertebrates holds the potential to gain a deeper understanding of infection transmission, which is the first step in a chain of enabling conditions that may cause virus spillover to humans (Plowright et al. 2015). Finally, a system that is applicable to small animals can also be deployed on other taxa such as reptiles or even large insects. The only limitation of the current system is that the individuals to observe need to be in proximity of a base station because of the need of synchronization between nodes. Consequently, observation is only feasible at repeatedly visited resting or mating sites or at foraging sites in species that are hunting locally and are philopatric.

### Outlook

A switch from traditional methods to the application of digital transceiver chips in proximity logging brings further advantages besides an enormous increase in data quality and quantity. The platform allows for the integration of additional sensors. For follow‐up models, we plan to integrate acceleration sensors that will decrease sampling rates of the proximity values during phases of inactivity in order to reduce energy consumption and extend battery life. Furthermore, animal‐borne sensors may locally store environmental and physiological data and transmit the data when the tag meets a ground node. A key development will be a wake‐up system that allows for communication between mobile nodes independently of ground nodes. This will enable the system to not only log encounters on a restricted focus area, but during the entire observation period without spatial restrictions. Finally, most variables such as sampling rates or signal range can easily be programmed and hence performance and life time can be tailored to a specific study species or an experimental design.

## Conflict of Interest

None declared.

## Supporting information


**Figure S1.** Hardware architecture of mobile node.

**Figure S2.** Hardware architecture of ground node.
